# Notes from Camp Sheridan

**Published:** 1918-01

**Authors:** 


### Notes From Camp Sheridan.

This covers an irregular area of about 3½ square miles, 2-3 miles N.E. of Montgomery, Ala. The soil is sandy, with deposits of gravel, and there is enough variation in grade to provide for free drainage by natural streams and ditches.

While an abundance of water could undoubtedly be obtained from wells, and of sufficient purity on account of the natural sand filters, the supply is taken from the city, which depends on Artesian wells. Electric current is also brought from the city. The surrounding country and, indeed, the camp site itself until taken over by the Government, is mainly occupied by negroes, living in small cabins, often with no windows. The condition of their premises is such that, in spite of sand filtration, most wells are very immediately contaminated or at least liable to contamination. Hence, all wells are placarded forbidden. The milk supply is also regarded as suspicious, and canned milk is used exclusively at present. There is no suspiciousness of sources of venereal infection. The proposition is a 99.40 approach to a sure thing. Hence, the only efficient means of avoiding infections of this nature are the reasonably prompt use of the modern prophylactic treatments, the restraint of soldiers already infected, if necessary by rigid confinement to detention camp and the activities of the military police—which have taken the place of the older temporary details under a provost martial. Any known danger point of any kind is guarded by the police. For instance a restaurant which did not conform to the necessary sanitary regulation, had police stationed at the doorway to prevent soldiers from entering—and it cleaned up. In regard to the venereal problem, it may well be that the general knowledge that infection is practically a sure thing—barring the result of prophylactic treatment—makes the actual incidence of cases less than with a small percentage of danger. And purely moral prophylaxis must be counted as an actual factor.

With a full division (approximately 25,000-30,000 men) there is bound to be a considerable aggregate of sickness and accident. Yet the theoretic mortality of such a body of men in peace, about 5:1,000 per year, has not been approached in the 2 or 3 months' experience. Probably the most frequent condition encountered is an exanthem, sometimes conforming to the condition of measles, sometimes to that of Rőtheln or German measles. Apparently more frequently than in peace, histories of recurrence and abortive and atypic cases are encountered. Such experience brings up the problem, already mentioned in this journal, of how diagnoses of exanthemata are to be absolutely established, and reminds us that, after all, we have very little scientific knowledge as to the identities and distinctions of the exanthemata. Practically, it is interesting to note that the old notion that exposure ''drove in'' measles, etc., or the more modern expression of the same idea to avoid criticism by the use of vaguer language, is not substantiated. In spite of inevitable exposure to cold in camp the mortality has been nil and practically all cases have been mild.

As nearly all men have been subjected to prophylactic vaccination against typhoid and the two para-typhoids, the Widal reaction is obviously excluded, since one would expect a positive reaction in practically all cases. Direct cultural methods, applied to faeces and urine, have been reduced to a practical basis, though the number of cases is very small. For instance, in a period of about a month, 12 cases suggesting typhoid occurred, but only 4 proved to be genuinely so and none were serious or protracted.

Pneumonia at one time seemed to threaten to become numerically serious, but a sudden cold snap checked the admissions and, at the same time, all the cases that were individually regarded as serious, improved. Possibly flies are the determining factor in conveying the infection. At any rate, one is impressed with the idea that while we used to think of pneumonia as a disease of cold and exposure, and while later, we used to put our tongue in our cheeks when we called it an infection and continued to regard it practically as a manifestation of "taking cold," it now appears frankly as an infectious general fever. At the same time, as a precautionary measure which seems to be of marked advantage, the line is following the advice of the medical staff in conducting the various drills so as to avoid chilling after perspiring, and in enforcing sleeping regulations that provide for dry, warm clothing, with sufficient ventilation.

Active tuberculosis—not, of course, in the sense of severe cases, but in the sense of warrantable diagnosis—has been found in 8-10 soldiers per 1,000 generally. The Ohio troops, formerly of the National Guard, comprising this division have a lower incidence, on account of selection from a good class of men and preliminary medical examination. The lowest incidence rate is held by a Utah regiment whose surgeon not only conducted a painstaking examination, but subjected every suspicious case to an advisory board.

It should be stated that active tuberculosis in the military sense, is quite a different conception from that generally held. Obviously, it is rarely that cases are admitted to service, which at this stage would show tubercle bacilli in the sputum or even furnish sputum in appreciable amounts. The tuberculosis board exists for the purpose of preventing such an eventuality even after months of exposure in the trenches. Percussion being often practiced where silence cannot be had, must be palpatory rather than audible, and fine discriminations of voice and breath sounds are impracticable. Hence, the method of Col. Bushnell for developing rales has been adopted as the best practical test for military purposes: Deep inhalation, full expiration terminating in a forced cough. Of course cases developing rales are subjected to closer study. Symptoms cannot be relied on as there is a temptation to exaggerate or misrepresent, sometimes to avoid, sometimes to secure military service. Misunderstandings also occur. For instance, haemorrhages are affirmed from nose bleed or accidental irritation of the pharynx, night sweats are construed to mean something entirely different. Cervical adenitis has been found almost universally, according to some, and is regarded as indicative of general, mild tuberculous involvement at an earlier age, healed before admission. The tonsils are also usually enlarged and ablation is encouraged. From some sections, most of the men have enlarged thyroids, rarely with symptoms. (Note: This is in keeping with Osler's observation in the valley of the Avon, and with the writer's in regard to Grand Island. There certainly is need of studying geology in connection with thyroid disease.)

The Tuberculosis Examining Board, from this recent experience with enormous numbers of normal men, has become sceptic as to the old notion that the right apex is normally relatively dull. In about 95% of all cases, no difference is noted, and the opinion is growing that the difference observed in civil practice is due to actual fibrosis. Only one cavity has been found in the entire division. The 1% or less of cases considered tuberculous are to be discharged, but it is hoped that some way may be devised to retain them under discipline for sanatorial treatment and eventual cure. Very few of these cases would show lesions by X-rays.

By a recent ruling, the radical operation for hernia is considered not dangerous to life and not necessarily involving a general anaesthetic. Hence along with haemorrhoids and enlarged tonsils, operation is compulsory and refusal subjects a man to court martial. However, some have doubted whether this would be the ultimate legal decision on appeal.

The medical men of the division, numbering somewhere between 150 and 200—there being frequent transfers—hold a society meeting every Friday at 7 p. m. Attendance is compulsory, unless one is engaged in more pressing duties or is excused.

That word compulsory suggests a needed explanation. Theoretically every thing in military life is compulsory. Many persons make themselves miserable over this fact, actually or in anticipation. The true philosophy can be acquired by considering one little item, about as absolutely compulsory as anything in military discipline, yet not laid down in any regulation. The priority of breeches over shoes in dressing is obligate, instead of facultate as in civil life. The raw recruit is likely to get the order reversed. If he is wise, he will accept the inevitable and learn how to dress from his first mistake. But there is a type of man who will tire himself by persisting in the attempt to dress the wrong way first, perhaps succeed in the attempt by tearing the knees out of his breeches. And there is another type who will immediately complain—more or less formally—and perhaps try to get the whole uniform changed.

It takes the first man only a few weeks to learn that he can dress more quickly than ever before, that he can keep warm—or cool—with fewer modifications of his garments, that his clothes are more economic than anything else he ever wore, that they will stand harder use and can be restored to a more presentable appearance more readily, that all sorts of button and suspender troubles are eliminated. In fact there is a reason for every little detail including the omissions.

The same general principle applies to almost everything else. There is a right way of doing almost everything, and there is a small percentage chance that the tyro will discover over the improvements that have been worked out, one item at a time, by many men of long experience. To return to the matter of medical meetings: It requires no argument that the surgeon ought to attend such a meeting and, as a matter of fact, it is a very pleasant duty. Also, the chances are about 9 out of 10 that if there were any really good reason why a man should be excused, even if the reason were purely personal or social, he would be. And, in these two humble illustrations, we have about the whole secret of adapting one's self to military discipline.

